# Bioformulation Containing Cohorts of *Ensifer adhaerens* MSN12 and *Bacillus cereus* MEN8 for the Nutrient Enhancement of *Cicer arietinum* L.

**DOI:** 10.3390/plants11223123

**Published:** 2022-11-15

**Authors:** Nitin Baliyan, Kamal A. Qureshi, Mariusz Jaremko, Minakshi Rajput, Monika Singh, Sandhya Dhiman, Dinesh Kumar Maheshwari, Chandra Kant, Ajay Kumar

**Affiliations:** 1 Department of Botany and Microbiology, Gurukula Kangri (Deemed to be University), Haridwar 249404, India; 2 Department of Pharmaceutics, Unaizah College of Pharmacy, Qassim University, Unaizah 51911, Saudi Arabia; 3 Smart-Health Initiative (SHI) and Red Sea Research Center (RSRC), Division of Biological and Environment Sciences and Engineering (BESE), King Abdullah University of Science and Technology (KAUST), Thuwal 23955, Saudi Arabia; 4 Department of Biotechnology, School of Applied and Life Sciences, Uttaranchal University, Dehradun 248007, India; 5 Department of Botany, Dharma Samaj College, Aligarh 202001, India; 6 Centre of Advanced Study in Botany, Institute of Science, Banaras Hindu University, Varanasi 221005, India

**Keywords:** endophyte, bacterial consortium, bioformulation, sugarcane straw ash (SCSA), soil fertility, plant growth

## Abstract

Here we examine the effects of different carrier based bioinoculants on the growth, yield and nutritional value of chickpea and on associated soil nutrients. A consortium of two taxonomically distinct endophytic bacteria—*Ensifer adhaerens* MSN12 and *Bacillus cereus* MEN8—have promising plant growth promoting (PGP) attributes. We demonstrate their delivery from the laboratory to the field via the formulation of an effective bioinoculant with economic and accessible carriers. Sugarcane straw ash (SCSA) was found to be an efficient carrier and bioformulation for enhancing viability and shelf-life of strains up to 12 months. A bioformulation containing an SCSA-based consortium (MSN12 + MEN8) increased seed germination by 7%, plant weight by 29%, length by 17%, seed-yield by 12%, harvesting index by 14% and proximate nutritional constituents by 20% over consortium treatment without SCSA. In addition, the bioformulation of post-harvest treated soil improved the physico-chemical properties of the soil in comparison to a pre-sowing SCSA-based bioformulation treated crop, being fortified in different proximate nutritional constituents including dry matter (30%), crude protein (45%), crude fiber (35%), and ether extract (40%) in comparison to the control. Principal component analysis and scattered matrix plots showed a positive correlation among the treatments, which also validates improvement in the soil nutrient components and proximate constituents by T6 treatment (MSN12 + MEN8 + SCSA). The above results suggest efficiency of SCSA not only as a carrier material but also to support microbial growth for adequate delivery of lab strains as a substitute for chemi-fertilizers.

## 1. Introduction

In agriculture, a large volume of agrochemicals including chemical fertilizers or chemical pesticides are applied to agricultural land to meet soil nutrient requirements or to control pathogens. However, the long term utilization of agrochemicals affects produce texture and nutrient yield, soil quality and the native microflora, as well as consumer heath [1]. Hence, there is an urgent need for an alternative to chemical fertilizers that can promote plant growth in a healthy way. On the other hand, ecological sustainability, decreased food security and limited field trials have opened a new horizon to revise strategies for the utilization of available carrier materials during the application of microbial inoculants. The selection of low-cost carriers for the formulation of bio-inoculants, which have the potential to support the growth of microbial strains, is still in its infancy [2]. In addition, the selected microbial strains must support the growth and development of plants via characteristic dominance in the rhizosphere.

In the last few years, various microorganisms have been utilized for the formulation of bioinoculants using myriad known carrier materials. In fact, bioinoculants are an eco-friendly and economically viable alternative to bacterial delivery in farming, but have so far not found space in the agro-market [3]. On the other hand, the nutrient status of soil and microbial diversity is being adversely affected by the reliance on agro-chemicals. It is, therefore, crucial to work on beneficial microorganisms to develop environmentally friendly strategies to enrich soil without the loss of microbial diversity and causing ecological harm. Beneficial bacteria have the capability to establish interactions with plants and improve plant health, biomass, and yield [4]. A formulation of bioinoculants uses local carrier materials suitable for as many bacterial species and strains as possible. The suitable supporting material must be non-hazardous, nontoxic, and have sufficient shelf-life [5]. Storage temperature and moisture content are important factors for agricultural products [6,7]. Hence, there is a need to re-look at alternative carrier materials that can be more beneficial and which can deliver the beneficial microorganisms from lab to land.

Chickpea (*Cicer arietinum* L.) is the most important winter (rabi) pulse crop and the oldest nutritious food of Asia [8]. An estimated 5.75 M tonnes of chickpeas holds a productivity of about 793 kg/h. The crop is currently growing in 7.58 M ha in India [9]. For this reason, the agricultural production of chickpeas should be more productive and sustainable. This communication aimed to investigate two diverse genera—*Ensifer adhaerens* and *Bacillus cereus*—in the growth promotion, yield and quality improvement of chickpea. We investigate using both combination and individual bioinoculant formulations in four affordably accessible and economically viable supporting materials used for the first time, namely sawdust, sugarcane straw, ash red-brick powder and field soil.

## 2. Materials and Methods

Sample collection of healthy chickpea plants was from a farmer’s agricultural field located in Muzaffarnagar (29°25′ N, 78°13′ E; 975 m; 819.2 ft.), Uttar Pradesh (India). The soil texture was determined by the sieve-method following the standard protocol [10]. However, the seeds of chickpea (*Cicer arietinum* L.) ver. PUSA-372-Desi were procured from the Govind Ballabh Pant University of Agriculture and Technology (University of Pantnagar), Uttarakhand, India.

### 2.1. Isolation and Characterization of Root-Nodulating and Non-Nodulating Endophytic Bacteria

The root-nodules and root-pieces (2 cm long) of the chickpea plant were used to remove adherent rhizospheric soil by washing and rinsing. The macerated nodules and root segments were plated on Yeast Extract Mannitol Agar (YEMA) and Luria-Bertani (LB) Agar media, respectively, following the serial dilution technique [11]. Next, the Petri-plates were incubated at 30 °C for 24–48 h until the growth of the bacterial colonies on the plate occurred. Following the growth of the bacterial colonies, these were purified by successive culturing to screen for different morpho-types. Beneficial bacterial strains were selected via a field-first strategy [12].

### 2.2. PGP Attributes of Beneficial Isolates and Venn Analysis

The PGP traits of the beneficial isolates were estimated using a standard protocol The Indole Acetic Acid (IAA) production test of the bacterial isolates was carried out following the protocol of Gordon and Weber [13]. Siderophore or iron-chelating (SID) tests were performed following the standard protocol of Schwyn and Neilands [14]. The potassium solubilization (KSH) test followed the protocol of Hu et al. [15] and the phosphate solubilization (PS) test followed the protocol of Pikovskaya [16].

### 2.3. Synergistic Interaction and Bacterial Consortium

The best suitable strain, which possesses PGP attributes, was selected for the consortia and evaluated for efficacy by growing together as per the standard protocol of Pierson and Weller [17] and Pandey and Maheshwari [18]. All nine strains were grown separately in the nutrient broth and then 100 μL of each culture was spread on the nutrient agar media (NAM) plates and incubated at 28 °C for 24–48 h. Following their growth, the isolates were restreaked for purification. However, for the bacterial consortium, a single colony of MEN8 and MSN12 was transferred in the fresh nutrient broth and incubated at 28 °C on the rotatory shaker at a speed of 120 rpm. Further growth was measured by taking the absorbance at 600 nm wavelength by using a UV-Vis Spectrophotometer (PG instruments, 3200, Lutterworth, United Kingdom). Further, 100 µL of active cultures (OD 0.6 at λ 600 nm) of MEN8 and MSN12 was transferred to 100 mL of fresh nutrient broth and allowed to grow synergistically to raise the consortium culture. The consortium culture was further subjected to measures of in vitro plant growth.

### 2.4. Molecular Characterization of Bacterial Isolates

The genomic DNA of the bacterial strains were extracted following the protocol of Tamura et al. [19]. The evolutionary relation of the strains [20] and the accession numbers were obtained from the NCBI GenBank Database.

### 2.5. Selection and Physico-Chemical Analysis of Supporting Materials

Sugarcane straw ash (SCSA), Brick-furnace red-brick powder (BFRP), Populus-Sawdust (PSD), Farmland Soil (FLS) and SCSA + FLS (1:1; *w*/*w*) were selected as test supporting materials. The test supporting materials were mixed in an equal volume (*w*/*v*) of distilled water and a paste was prepared with thorough stirring. The pH was determined in a 1:2.5 (*w*/*v*) ratio by using a digital flat bottom pH meter. The moisture content (MC) was measured on the basis of wet and dry weight, each 100 g of oven dried (for 24 h on 40 °C) supporting material was stirred in a 500 mL beaker having 200 mL of water. Saturated material was decanted on gauze for 1 h to eradicate extra water to obtain a paste suspension. The remaining mass of the supporting material was observed and the percent water holding capacity (WHC), or other various parameters were calculated according to Rodrigues and Rodrigues [21].

### 2.6. Preparation of Bioformulation

The method described by Namsena et al. [22] was adopted and modified for the preparation of the bioformulation. The supporting materials were dried under shade and grinded as an amorphous powder. A total of 50 g of each processed material was water-soaked in sterilized distilled water (SDW) for 24 h and kept in an autoclaved 500 mL conical flask. The pH of the supporting materials was brought to neutral by adding CaCO_3_ after sterilization over three successive days, which aids in distinctly preparing inoculants as per the guidelines of the Bureau of Indian Standards [23]. The quality and sterility of the supporting materials on nutrient agar plates was checked via the serial dilution method. The bacteria were grown in LB and YEM broth up to the attainting maxima of log phase (24 h and 20 h, respectively) and the culture biomass was harvested at 10,000 rpm for 20 min at 4 ± 1 °C using centrifugation (Centrifuge TC-4109 Elektrocraft). The equivalent biomass of cell pellets from each bacterial culture and consortium culture (able to raise the initial bacterial count 10^8^ cfu/g by dissolving in 1000 mL distilled water) were supplemented to reach 1000 g (*w*/*w*) of sterilized supporting materials. After thorough mixing using a mixer, they were spread onto sterilized trays (25 × 18 × 5 cm). The shallow-tray materials were then transferred to sterile HiMediaPolybags™, (Mumbai, India) aseptically to prevent desiccation. The low density polyethylene (LDPE) polybags (leaving about 70% vacant space) were punctured on the lateral sides to provide enough aeration to the bioinoculants. For curing, the polybags were incubated at 25 ± 1 °C temperature for 48 h in a BOD incubator (Bionics Scientific Technologies, BST/BOD-173, Delhi, India) and stored at room conditions for 365 days at ambient temperature. In the control bioformulation, only sterile carrier materials were used to process and incubate for the equivalent duration and in similar conditions. Each process of the bioformulation was performed in triplicate.

### 2.7. The Viability and Shelf Life of Bacterial Strains

The viability and shelf-life of the supporting materials was assessed through a bacterial population count up to 12 months. The samples of different bioformulations were collected during different time intervals aseptically. The bacterial populations were determined by suspending 1.0 g bacterial inoculant into 9.0 mL of 0.9% sterile normal saline (SNS) solution (*w*/*v*) comprising 0.1% Tween-80 and then serially diluted up to 10^−6^ and 10^−8^ dilutions. A 100 µL inoculum of these dilutions was spread on the YEMA and LB agar plates and incubated at 30 °C for 24–48 h for the growth and enumeration of the bacterial populations. Further bioformulations were made following the standard protocol mentioned by Biradar and Santhosh [24].

### 2.8. Field Experiments

The field study was conducted at Village Karwara (29°25′00″ N, 77°35′12″ E; 249 m MSL), Muzaffarnagar, India from October 2016 to February 2017. The study area is semi-arid, with a moderate winter and an average seasonal temperature ranging from 7.4 °C minimum to 31.9 °C maximum and with an average annual rainfall of 890 mm (average data of 2016–2017; source of data: AccuWeather). We developed six treatments, T1 to T6 using SCSA as the base to carry the bacterial cultures MSN12, MEN8 and the consortium MSN + MEN8. Seeds without treatment served as a control (C). A random plot design was used for sowing the bio-primed seeds with supporting material in a 1 m^2^ area (in 6 rows, 20 cm apart, having 11 seeds each at 10 cm apart) with three replicates of each treatment. A recommended dose of 2 kg acre^−1^ of prepared different bioformulation was added to the treated plots before sowing the seeds [25]. Before sowing, the seeds were surface sterilized under aseptic conditions using NaOCl (2%) for 30 min, followed by twice washing with ethanol (90%) for 5 min, and three washes with sterilized distilled water. The seeds were then soaked in the two months old bioformulation mixture (9 × 10^8^ cfu g^−1^) and sterile distilled water (1:1, *w*/*v*).

After 2 h of soaking, the coated seeds were air dried in the shade for 30 min and 66 seeds were sown in each experimental plot to evaluate the germination test. Chemical fertilizers were not added to the field during the course of study. After sowing, the germination parameters were observed and recorded daily from day 3–15 after sowing. The plants were uprooted to measure plant weight fresh/dry and height root/shoot at 30 DAS and seed yield/harvesting index at 120 DAS. Plant growth along with yield parameters were recorded according to Baliyan et al. [11].

### 2.9. Physico-Chemical Properties of Pre-Harvesting and Post-Harvesting Soil

The aim of pre- and post-harvested soil sample analysis was to determine the effects of the bioformulation on soil fertility and the available mineral content. Briefly, the MC (moisture content) and pH were examined following the protocol of Kumar et al. [26], while the mineral content, such as Avl. (available) N [27], Avl. P [28], Avl. K [29], along with OC [30], were also determined using standard protocols.

### 2.10. Proximate Composition Analysis

After harvesting, the seeds were collected from each treatment group and used to evaluate the proximate composition. An evaluation of dry matter, crude proteins, total ash, moisture, acid insoluble ash, ether extract and crude fiber contents in the seeds of chickpea was performed following the methods defined by Nobile et al. [31]. A component analysis was carried out to determine the statistical correlation between the different treatments and pre- and post-harvested soil nutrients (N, P and K) by using principal component analysis software.

### 2.11. Statistical Analyses

All the data were collected in triplicate to analyze the statistics. A Venn diagram was constructed to evaluate the PGP attributes shown by the bacterial isolates using VENNY 2.0 software *(*http://bioinfogp.cnb.csic.es/tools/venny/ accessed on 15 June 2022). However, the regularity and homogeneity tests of variance were tested and the average variance in the shelf-life of the bioformulations and vegetative plant growth parameters were evaluated using Duncan’s multiple range test (DMRT) and the analysis of variance (ANOVA) at (*p* < 0.05) by using a statistical package for the social sciences (SPSS) version 20.0 (Chicago, IL, USA) software. A seed yield and harvesting index were conducted by plotting a boxplot with five representative values of each treatment showing the mean value (Q3) and standard deviation (scattered lines) by using plotly software. Pair-wise scatter plots, a visualization of variables, and the correlation among the variables in a matrix setup were performed using Origin (Origin software version 20, Northampton, MA, USA).

## 3. Results

The soil texture was Udic Haplustepts (Great-group: Haplustep; Sub-group: Udic) with a different color on account of numerous edaphic factors. Out of 39 bacterial isolates, based on their colonial variations and generation time, nine putative isolates nodulating *Rhizobia* and four non-nodulating endophytic *Bacillus* isolates were selected from chickpea nodules and root tissues, respectively.

### 3.1. PGP Characterization, and the Identification of Endophytism

**Figure 1:**
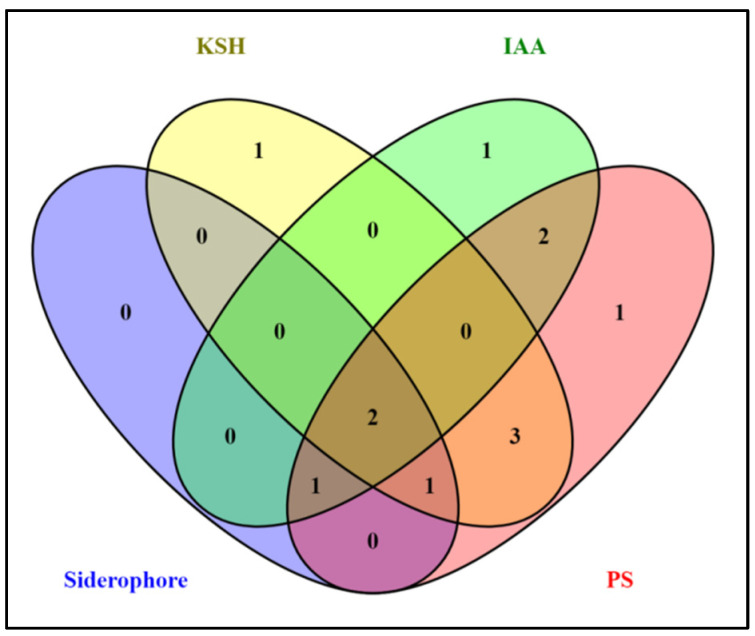
Venn diagram showing the plant growth promoting attributes of bacterial isolates. PS: phosphate solubilization; IAA: indole acetic acid production; SID: Siderophore production; and KSH: potassium solubilization.

Figure 1 shows a Venn diagram of different PGP traits or parameters such as IAA production, iron-chelating (SID) or siderophore production, potassium solubilization (KSH) and phosphate solubilization (PS). This displays a logical relationship among the isolates. Among all 13 isolates (including both nodule and root endophytes), 10 isolates were positive for phosphate solubilization, 7 for potassium, 4 showed siderophore secretion and 6 produced IAA (Figure 1). Out of the 13 isolates, only MSN12 and MEN8 showed all PGP characteristics maximally, as observed with qualitative and quantitative analysis.

Among the 13 isolates and their pairs, MSN12 and MEN8 coupled their PGP attributes with no zone of inhibition during in vitro synergistic interaction and showed compatibility between each other. They were, therefore, considered a consortium for further study. The effect of co-inoculation was studied in terms of the growth rate of MSN12 and MEN8. The *K*-value of MSN12 and MEN8 were 3.73 ± 1.2 and 2.15 ± 0.4, respectively, in single species culture. In the mixed culture, MEN8 induced a K-value of MSN16 up to 4.23 ± 0.8, which was 11.82% more than that of individually grown cultures, while the K-value of MEN8 was 2.23 ± 0.3 in the consortia, which was 3.58% more than in individual cultivation.

In vitro study showed the potential of two isolates—MSN12 and MEN8—in fixing nitrogen and IAA secretion in the range of 76.2–81.4 µg/mL. Consortia of these two strains showed a higher secretion of IAA 83.7 µg/mL after 168 h. Individually, the strain MEN8 produced 5% IAA more than MSN12. Similarly, the phosphate solubilization efficiency (PSE) of MSN12 and MEN8 were recorded at 52.6 and 62.8%, respectively. Similarly, both MSN12 and MEN8 strains also solubilized potassium (K) in the order of 46.5 and 49.3% KSE (K solubilization efficiency), respectively. However, the solubilization efficiency of both P and K was <52–63% in the consortia (MSN12 and MEN8) in comparison to the individual isolates. The individual isolates and consortium of MSN12 and MEN8 secrete 43.17, 44.23 and 44.85% siderophore, respectively. The production of volatile cyanogen (HCN) was also demonstrated in these bacterial isolates individually as well as in the mix-culture. Molecular 16S rRNA gene sequencing and phylogeny analysis confirmed that strain MSN12 was *Ensifer adhaerens* whereas MEN8 was identified as *Bacillus cereus*. The gene sequences of both strains were deposited in the NCBI under accession number MH613070 and MH613068, respectively (latest access 5 July 2022).

### 3.2. Supporting Materials

Physico-chemical studies of the supporting materials showed that SCSA contained the maximum inherent moisture content followed by PSD, SCSA + FLS, FLS and BFRP. SCSA had neutral pH 7.0 and fulfilled the most suitability criteria as a carrier material for augmentation of active bacterial growth. This also distinguishes the growth behavior of bacteria compared to their water holding capacity. This property helps the bacterial isolates in providing good aeration support during their growth in supporting material-based preparations; other properties of supporting materials are shown in Table 1.

### 3.3. Determination of Shelf-Life of MSN12 and MEN8 Strains on Different Supporting Materials

**Figure 2:**
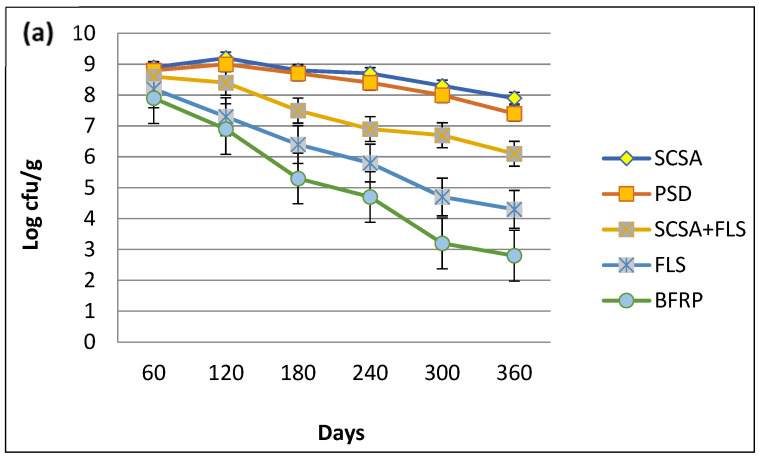

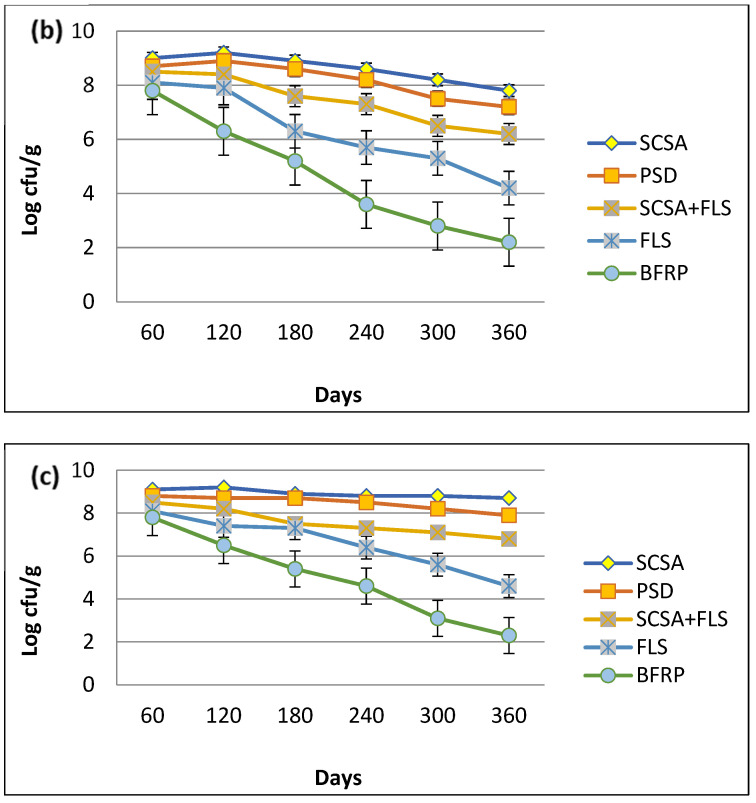
Shelf-life assessment of *E. adhaerens* MSN12 (**a**), *B. cereus* MEN8 (**b**), and the consortia of MSN12 + MEN8 (**c**), in different supporting materials. CFU g^−1^ values were log-transformed (log CFU g^−1^) to improve the homogeneity of variances and then statistical comparisons were performed using ANOVA and the Duncan’s multiple range test, with significance at *p* < 0.05.

The shelf-life of MSN12 and MEN8 in bioformulations was studied over different time intervals. Among all of the bioformulations, SCSA was the most suitable supporting material for both *E. adhaerens* MSN12 and *B. cereus* MEN8 (Figure 2) since they were present up to 6.81 × 10^8^ and 6.33 × 10^8^ cfu/g, respectively, after 120 days of stability at room temperature. On the other hand, both strains supported the maximum population density up to 90 days in soil + ash-based preparations, but the bacterial populations drastically declined up to 3.27 × 10^7^ and 4.08 × 10^7^ cfu/g for MSN12 and MEN8, after 180 days of stability (Figure 2a–c). However, it was also observed that co-inoculation preparations of both *E. adhaerens* MSN12 and *B. cereus* MEN8 were higher (11%) than the respective individual strains. On the other hand, the red-brick powder and sawdust proved inferior in the study to that of *E. adhaerens* and *B. cereus* as evidenced by 2.34 × 10^9^ cfu/g and 6.94 × 10^9^ cfu/g, respectively, after 180 days of stability.

### 3.4. Field Studies

#### 3.4.1. Soil Analysis before Sowing

The pre-harvested soil was sandy and loamy in texture with sand (72–75%), silt (12–14%), and clay (8–11%). The pre-harvested soil examined had the following properties: MC: 9.68 ± 0.07%; pH: 6.9 ± 0.10 (1:2.5; *w*/*v*); Organic carbon (OC): 0.93 ± 0.02%; available Phosphate (Avl. P): 17.65 ± 0.09 kg ha^−1^; Avl. Nitrogen (N): 263.15 ± 15.28 kg ha^−1^; Avl. Potassium (K): 293.45 ± 17.38 kg ha^−1^.

#### 3.4.2. Effects on Seed Germination, Growth and Yield of Chickpea

The SCSA-based bioformulated treated plants induced vegetative and reproductive growth and development of *C. arietinum* in field experiments. Co-inoculation of both MSN12 and MEN8 strains with SCSA significantly increased (*p* > 0.01) seed germination (up to 15 DAS), as well as vegetative parameters, such as plant weight fresh/dry and root/shoot length as recorded at 30 days of sowing (DAS) in both of the years 2016–2017 and 2017–2018 compared to the control (Table 2). T6 displayed the greatest seed germination (73%) over the control among all of the treatments. The average root-length and shoot-length were maximal in T6, which was 65% and 79% more than the respective controls, followed by the plant raised after T4. The maximum fresh-weight of the plant was enhanced 91% over the control by T6. Plant dry-weight increased maximally in all of the treatments over their respective non-bacterized control (Table 2).

**Figure 3:**
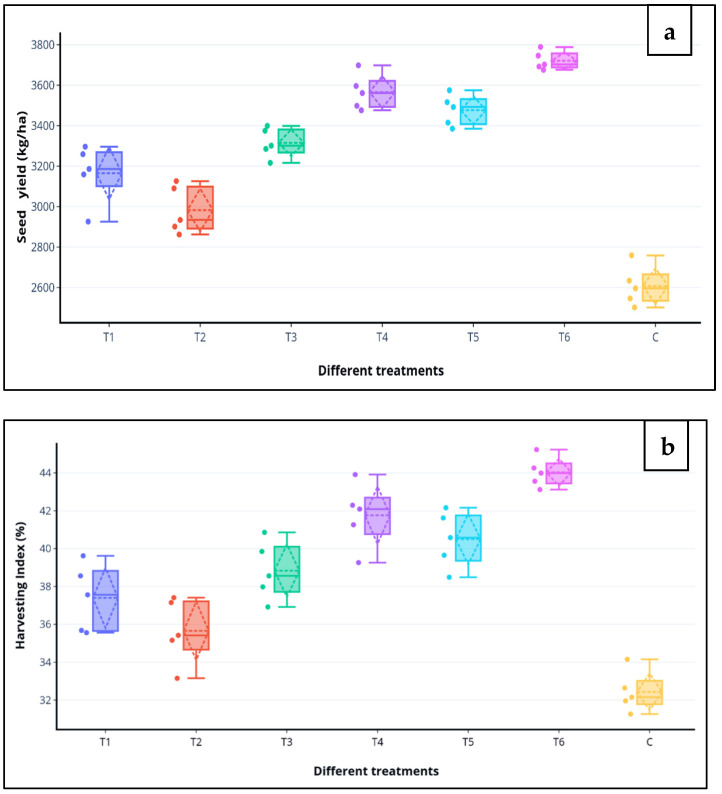
Effects of various treatments groups on the (**a**) seed yield (kg/ha) and (**b**) harvesting index (%) of chickpea in the field after 120 DAS. Boxplot representing five values of each treatment and showing the minimum value by the lower quartile (Q1—0.25), and the maximum value by the upper quartile (Q3—0.75). The horizontal line drawn in the middle denotes the median (Q2—0.50) and scattered lines show the standard deviation.

During the 2016–2017, treatment, T6 (MSN12 + MEN8 with SCSA) showed seed yield up to 36% and harvest index 26%, while during 2017–2018, the same treatment showed an increase in seed yield up to 40% and harvesting index 32% over the control. The mean value of seed yield and harvesting index of the two years showed an increase in comparison to the non-treated control (Figure 3a,b).

### 3.5. Correlated Study of Pre- and Post-Harvested Soil via Principal Component Analysis (PCA)

**Figure 4:**
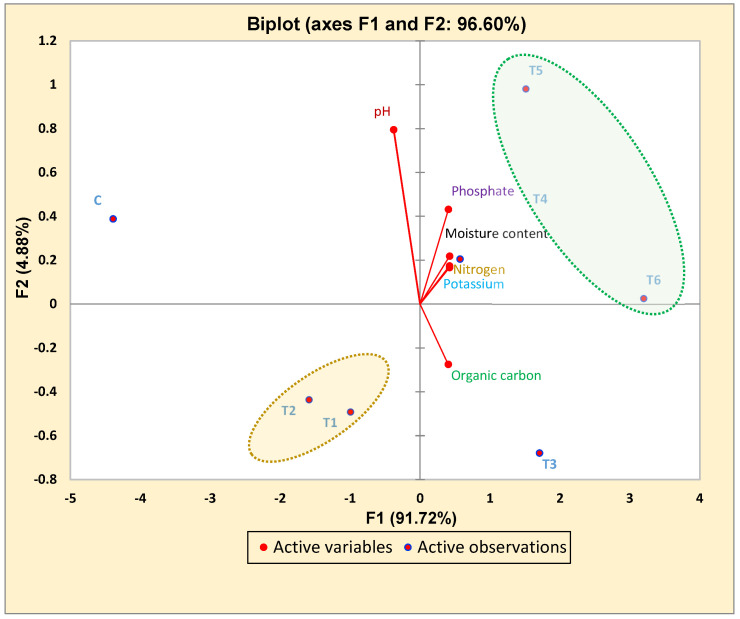
PCA analysis showing the correlation between the different treatment groups and variables of soil nutrients.

The soil pH was observed before and after the harvesting of crops and was slightly acidic after harvesting. Other properties, such as the average Avl. N, Avl. P, Avl. K, MC, and OC were also measured in post-harvesting soil and a significant increase of 23%, 88%, 37%, 47% and 22%, respectively, was observed in the T6 treatment as compared to the control soil. A PCA was used to determine the statistical correlation between the different treatments and the pre- and post-harvested soil nutrients. Different soil nutrients are explained with the component PCA1: 63.9% and component PCA2: 20.1% (Figure 4). A PCA showing the correlation between different treatments and different soil nutrients. [Where, T1 (MEN8); T2 (MSN12); T3 (MSN12 + MEN8); T4 (MEN8 + SCSA); T5 (MSN12 + SCSA); T6 (MSN12 + MEN8 + SCSA); C (without bio-inoculant and carrier); and Nitrogen (kg ha^−1^), Phosphate (kg ha^−1^), Potassium (kg ha^−1^), Organic carbon (%); Moisture content (%)].

The PCA analysis showed that treatment groups T4, T5 and T6 were highly effective in enhancing soil nutrients (N, P and K) as evidenced by their positive correlation, as well as their presence in the same group. However, the treatment group T3 was present in a similar domain and was positively correlated with the enrichment of organic carbon. The treatment groups T1 and T2 showed less of a contribution from these treatments in the enhancement of soil fertility and were negatively correlated, as shown in Figure 4.

### 3.6. Correlation Study of Proximate Components via Scatter Matrix Visualization

**Figure 5:**
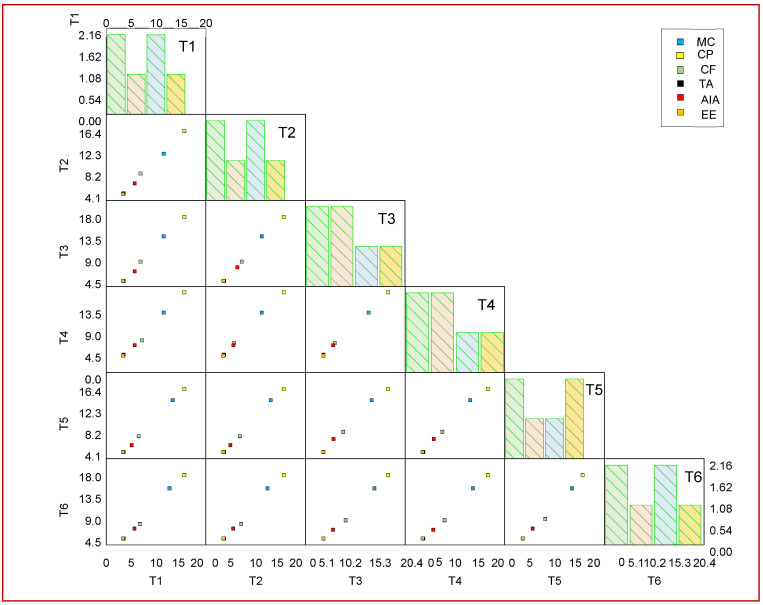
Scatter plots of variable correlation displayed in a matrix for different treatments and proximate constituents. Values are expressed as mean ± SD (n = 5) with a level of significance at *p* < 0.05. Abbreviations: DM: Dry matter; Moist: Moisture; CP: Crude protein; CF: Crude fiber; TA: Total ash; AiA: Acid insoluble ash; EE: Ether extract. T1 (MEN8); T2 (MSN12); T3 (MSN12 + MEN8); T4 (MEN8 + SCSA); T5 (MSN12 + SCSA); T6 (MSN12 + MEN8 + SCSA).

Proximate analysis showed that treatment with the *Bacillus* and *Ensifer* species improved the chemical constituents in the seeds of chickpea. Treatment T6 (MSN12 + MEN8 with SCSA) was found to be the most superior among all of the treatments in boosting the chemical constituents. A maximum increase in ether extract of 41% was recorded in T6 relative to the control, followed by T4 alone treated plants, which were 5% lower in comparison to T6. The consortia of MSN12 and MEN8 with SCSA (T6) was found to increase the contents of crude fiber by 35% in the plants followed by T4, which was 18% lower in comparison to the consortia and 30% higher in comparison to the control. Dry matter was 30% more improved by treatment T6 followed by T3, which was 7% lower in comparison to treatment T6. The proximate component data were analyzed through a scatter matrix plot, showing variation in the constituents triggered by different treatment groups (Figure 5).

## 4. Discussion

Nowadays, endophytic microorganisms are gaining momentum in sustainable agriculture due to their superior colonization efficacy and capability to ameliorate biotic and abiotic stresses [32,33]. However, the selection of suitable supporting materials for bacterial inoculant preparation remains challenging [34]. For this reason, assessment of inoculated bacteria in supporting materials is one strategy for the enhancement of crop productivity [35]. In this study, PGP *Ensifer adhaerens* MSN12 and *Bacillus cereus* MEN8 exhibited their inherent potential to synthesize IAA. Earlier, some other bacterial genera (*Bacillus, Rhizobium, Pseudomonas, Ensifer,* etc.) were also reported to support IAA production [3,12,36]. Mishra et al. [37] reported rhizospheric isolates with the potential to produce IAA, which after application, significantly enhanced plant growth and also maintained soil nutrient quality. In our study, both the strains MSN12 and MEN8 solubilized phosphate (P) and potassium (K) salts to improve P and K availability to the growing chickpea. Various mechanisms have been established for insoluble P solubilization in the soil by rhizospheric *Ensifer* and *Bacilli* strains as observed by Meena et al. [38]. In addition, MSN12 and MEN8 strains produced siderophores that individually favor the growth of the chickpea, as also observed by Arora et al. [39]. Similarly, HCN produced by MSN12 and MEN8 benefited chickpea plant growth as an added advantage. In our study, the consortia of bacterial strains exhibited better PGP influence under field conditions. In previous studies, various authors have shown the significance of microbial consortia in plant growth [11,18]. Both of the strains grew synergistically in vitro, perhaps due to their better utilization of a shared nutritional niche as also observed by Pandey and Maheshwari [18].

It is challenging to maintain the viability of bacterial cells for long term storage and potency, due to limitations of nutrient contents [40]. Such issues have hampered the commercialization of bacterial inoculant preparations. We observed that sugarcane straw ash-based supporting material was the most suitable, since it is economically viable, eco-friendly, and easily available. Various supporting materials have been evaluated for their ability to keep microbes viable for longer, reducing desiccation and enhancing their adhesion to plant parts [2]. In our study, a neutral pH of 7.0 and pore spaces in SCSA sustain the number and viability of bacteria at higher levels in comparison to that of other supporting materials [41]. In a detailed study Brockwell and Bottomley [42] reported that, to support the growth of a large microbial population, the supporting materials must possess high WHC with good aeration and have a near to neutral pH. Likewise, the high availability of macro and microelements in SCSA act as immediate precursors of certain nutrients, such as N, P, K, Mn, low Mg and Fe elements, to enhance the survival of both *E. adhaerens* MSN12 and *B. cereus* MEN8. The protective properties of SCSA may be attributed to its capacity to maintain ideal water availability for bacterial survival and its ability to develop interactions amidst seed and bacterial cells. Independently, two groups of scientists [41,43] observed the effect of burning and trashing on sugarcane leaf analyses. The dual advantage of SCSA was effective in favoring soil properties because of its inherent absorption of moisture content, water holding capacity, optimum pH, non-toxic nature, and ease of use to enhance the growth and yield of chickpea. Recently, Paliya et al. [44] used sludge ash as a carrier and proved its role in plant germination enhancement. Although authors have reported enhanced survivability of bacterial genera on various supporting materials [5,45], the continued existence of *Ensifer* and *Bacillus* in supporting materials seems to be a novelty. Thus, the shelf-life of the strains *E. adhaerens* MSN12 and *B. cereus* MEN8 proved that SCSA is the most suitable supporting material (carrier), followed by PSD and FLS for *E. adhaerens* and *B. cereus* preparations.

In this work, a potent bioformulation increased the mineral content of soil significantly (paired sample *t*-test, *p* < 0.05) and improved its fertility as proved by soil analysis after crop harvest. A suitable pH for plant growth in a treated field is slightly acidic. Organic carbon and moisture content was also found to be enhanced in the treated plots, with similar findings by Kushwaha [46]. Earlier, Chinnusamy et al. [47] reported that the application of biofertilizers considerably improves mineral content and that bioformulations significantly increase N, P and K and other mineral content in soil, which assists in plant growth and, thereby, improves yield. The PCA data displayed a positive correlation among the different treatment groups with different nutrient contents in the soil, which confirms the enhancement in the soil’s nutrient content by the utilization of these bacterial strains formulated with SCSA. Thus, the data favored T4, T5 and T6 as the best treatments for soil nutrient enhancement and increased soil fertility. Enhancement with maximum proximate chemical constituents was recorded with SCSA containing both MSN12 and MEN8. Pandey et al. [3] reported crude proteins and carbohydrate content increase due to *Bacillus* treatment in the grains of *Amaranthus hypochondriacus.* The results obtained in the present study show that *E. adhaerens* and *B. cereus* have several merits for chickpea plant growth. The use of SCSA supporting material for both *E. adhaerens* and *B. cereus* inoculant preparation undoubtedly showed advantages over traditionally used seed dressing besides facilitating delivery and handling.

## 5. Conclusions and Perspectives

In the current situation of global climate change, there is a need to recognize a sustainable method of agriculture. In this context, the utilization of microbial consortia is an emerging prospect to enhance agricultural yield. In our study, SCSA significantly (*p* < 0.05) enhanced shelf-life and supported the growth of both the strains *E. adhaerens* MSN12 and *B. cereus* MEN8. Both strains showed a positive response in terms of plant growth. The bioformulation or consortia of SCSA and *E. adhaerens* MSN12 and *B. cereus* MEN8 showed a significant enhancement in seed germination, plant growth, yield, and soil fertility. Thus, the tested bioformulation has the potential to improve agricultural yields and can be used at the industrial level to deliver an ample number of microbes for chickpea cultivation by replacing chemical fertilization. On the basis of these studies, SCSA could be used as a potential carrier material in sustainable agriculture systems. An effective bioformulation can be prepared for effective commercialization by using other waste products, and this will need further studies to support agronomical improvement.

## Figures and Tables

**Table 1:** Physico-chemical properties of different supporting materials.

Physical Parameters	PSD	SCSA	FLS	SCSA + FLS	BFRP
Moisture content (%)	11.6 ± 0.01	12.5 ± 0.01	3.7 ± 0.01	8.3 ± 0.01	0.9 ± 0.01
% Water holding capacity (WHC)	76 ± 1.46	89 ± 1.51	68 ± 0.45	72 ± 0.93	42 ± 0.35
pH [1:2.5 (*w*/*v*)]	6.2 ± 0.01	6.8 ± 0.01	6.9 ± 0.01	7.2 ± 0.01	6.0 ± 0.01
Chemical oxygen demand (COD) (mg/L)	25.4 ± 0.92	32.4 ± 0.98	16.5 ± 0.92	21.3 ± 0.91	11.5 ± 0.90
Biological oxygen demand (BOD) (mg/L)	20.6 ± 0.85	28.4 ± 0.86	22.1 ± 0.72	19.6 ± 0.71	14.4 ± 0.68
Total carbon (%)	18.5 ± 0.68	24.8 ± 0.72	17.3 ± 0.65	15.4 ± 0.67	12.6 ± 0.61
Total nitrogen (%)	1.4 ± 0.01	1.5 ± 0.01	1.1 ± 0.01	1.1 ± 0.01	0.06 ± 0.00

Values are the mean replicates (mean ± SE). Abbreviations: PSD: *Populus*-sawdust; SCSA: sugarcane straw ash; FLS farmland soil; BFRP: brick-furnace red-brick powder.

**Table 2:** Effects of different treatments on seed germination and vegetative parameters of chickpea during field assessment (mean value of two years 2016–2017 and 2017–2018).

Different Treatments	SG (%) ^a^	Plant Weight (g) ^b^		Plant Length (cm) ^b^	
		Fresh	Dry	Root	Shoot
T1 (MEN8)	90.9 **	3.37 *	0.89 **	11.2 **	23.4 **
T2 (MSN12)	88.1 **	3.14 *	0.81 **	10.2 **	19.3 **
T3 (MSN12 + MEN8)	91.4 **	4.24 *	1.17 **	13.7 **	25.3 **
T4 (MEN8 + SCSA)	94.6 **	4.79 *	1.35 **	14.6 **	24.9 **
T5 (MSN12 + SCSA)	92.2 **	4.38 *	1.23 **	13.5 **	23.2 **
T6 (MSN12 + MEN8 + SCSA)	97.8 **	5.42 *	1.58 **	16.2 **	29.6 **
C (without bio-inoculant and carrier)	56.5	2.83	0.59	9.8	16.5
SEM	1.7	0.63	0.12	0.61	0.72

Values are the mean of five plant samples from each treatment and data are significant at *p* < 0.05 with ANOVA; ** = significant *p* < 0.01 in LSD; * = significant at *p* < 0.05 in the LSD as compared to control. Abbreviation: MSN12: *E. adhaerens*; MEN8: *B. cereus*; SG: seed germination; C: control; SEM: standard error of the mean; a: 15 DAS (days after sowing); b: 30 DAS (days after sowing).

## Data Availability

Not applicable.
